# Supplementary figures and images for: Diuron tolerance and potential degradation by pelagic microbiomes in the Great Barrier Reef lagoon
Source: PeerJ. 2016 Mar 8;4:e1758. doi: 10.7717/peerj.1758 (PMC4793316; doi:10.7717/peerj.1758)

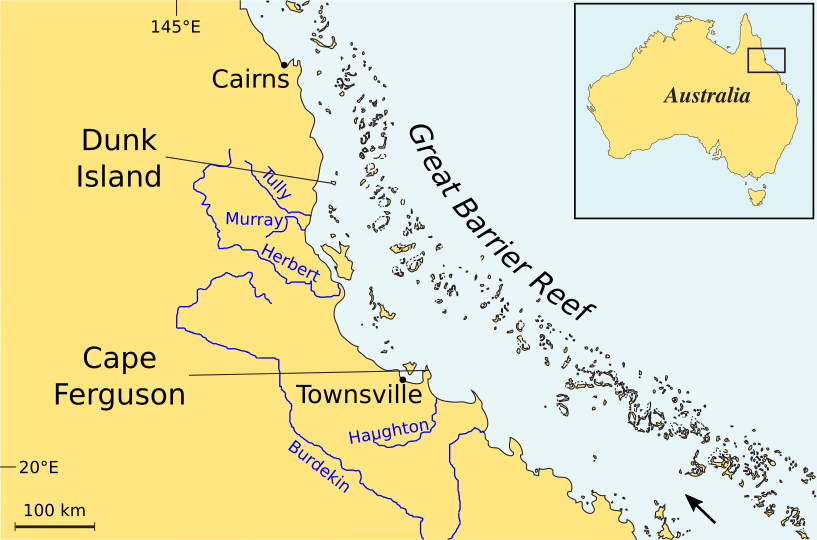

Supplement: Figure S1 — The sampling locations within Australia (inset) and the Great Barrier Reef lagoon (main panel) are shown. The main rivers influencing these sites are depicted in blue and the arrow indicates the direction of the main current. [file peerj-04-1758-s001.png]

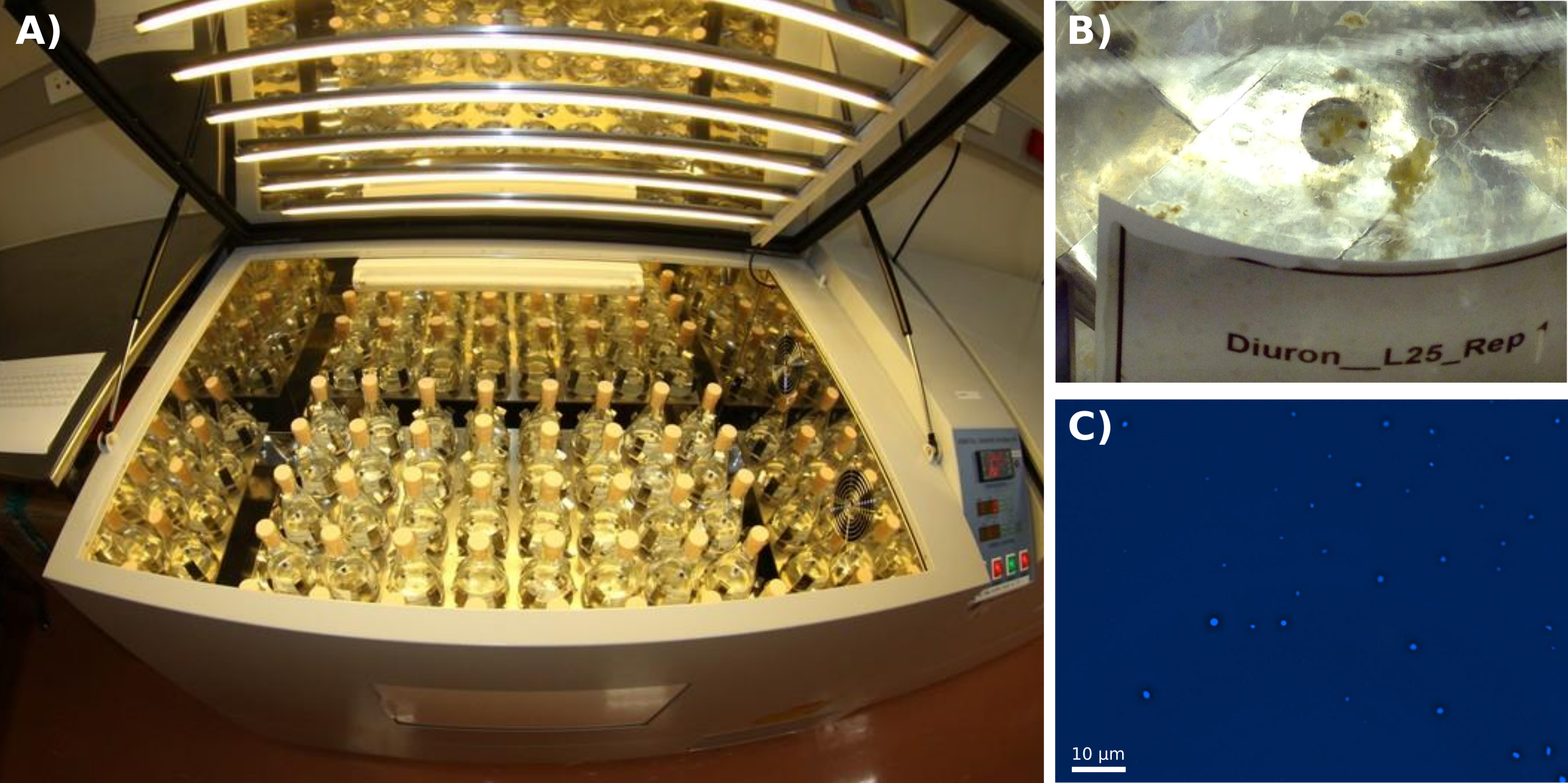

Supplement: Figure S2 — (A) Triplicate flasks incubated at 25 °C with or without diuron amendment, and with or without light for 365 days. (B) Example of a flask incubated with diuron in the light and in which signs of growth are visible at day 365. (C) Micrograph showing DAPI-stained cells in a diuron-treated light-incubated flask collected at day 150. [file peerj-04-1758-s002.png]

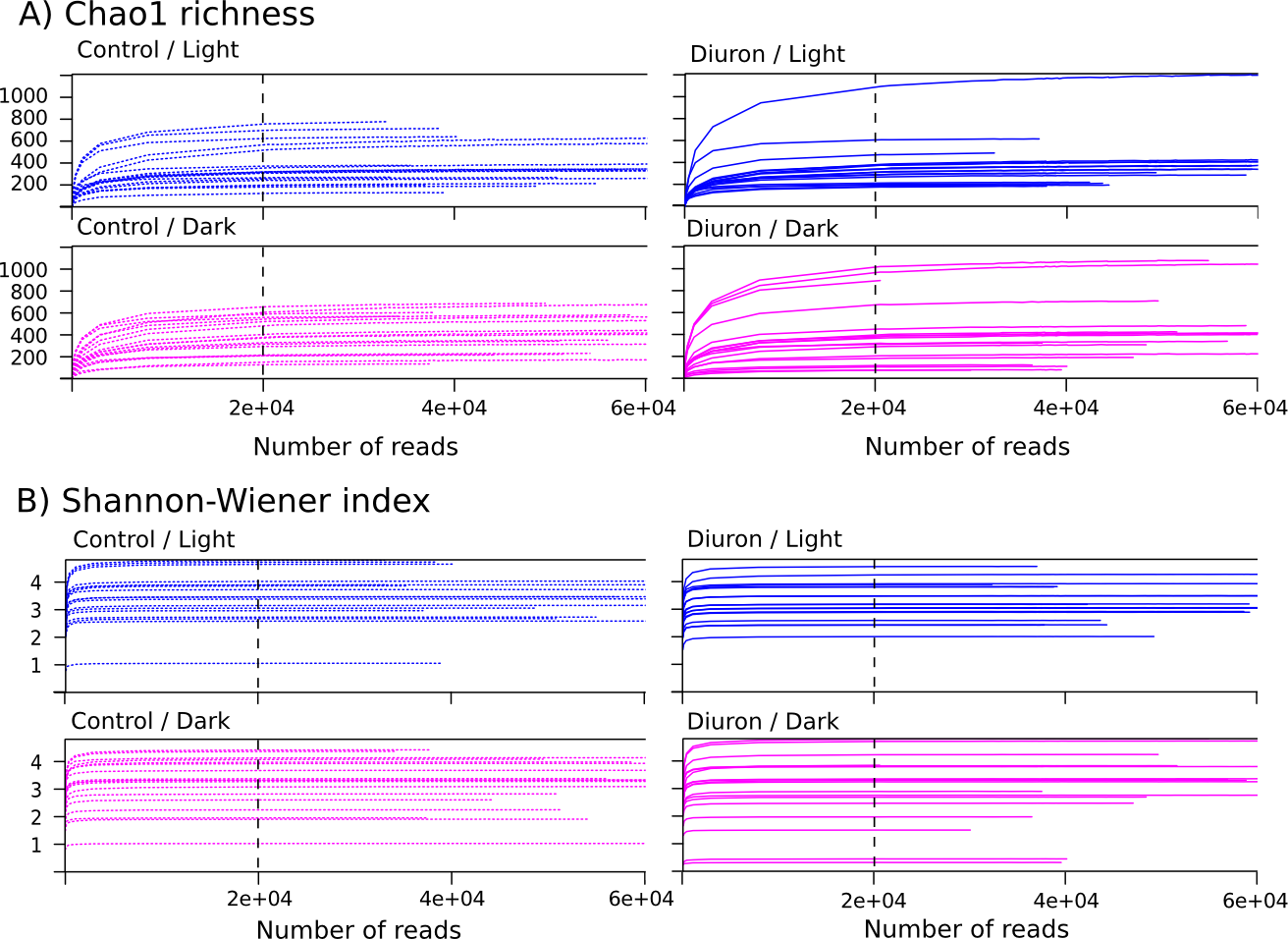

Supplement: Figure S3 — These rarefaction curves show sample OTU diversity as a function of sequencing depth for each experimental treatments: (A) Chao1 richness and (B) Shannon-Wiener index. The black dashed line indicates the rarefaction depth used in this study. [file peerj-04-1758-s003.png]

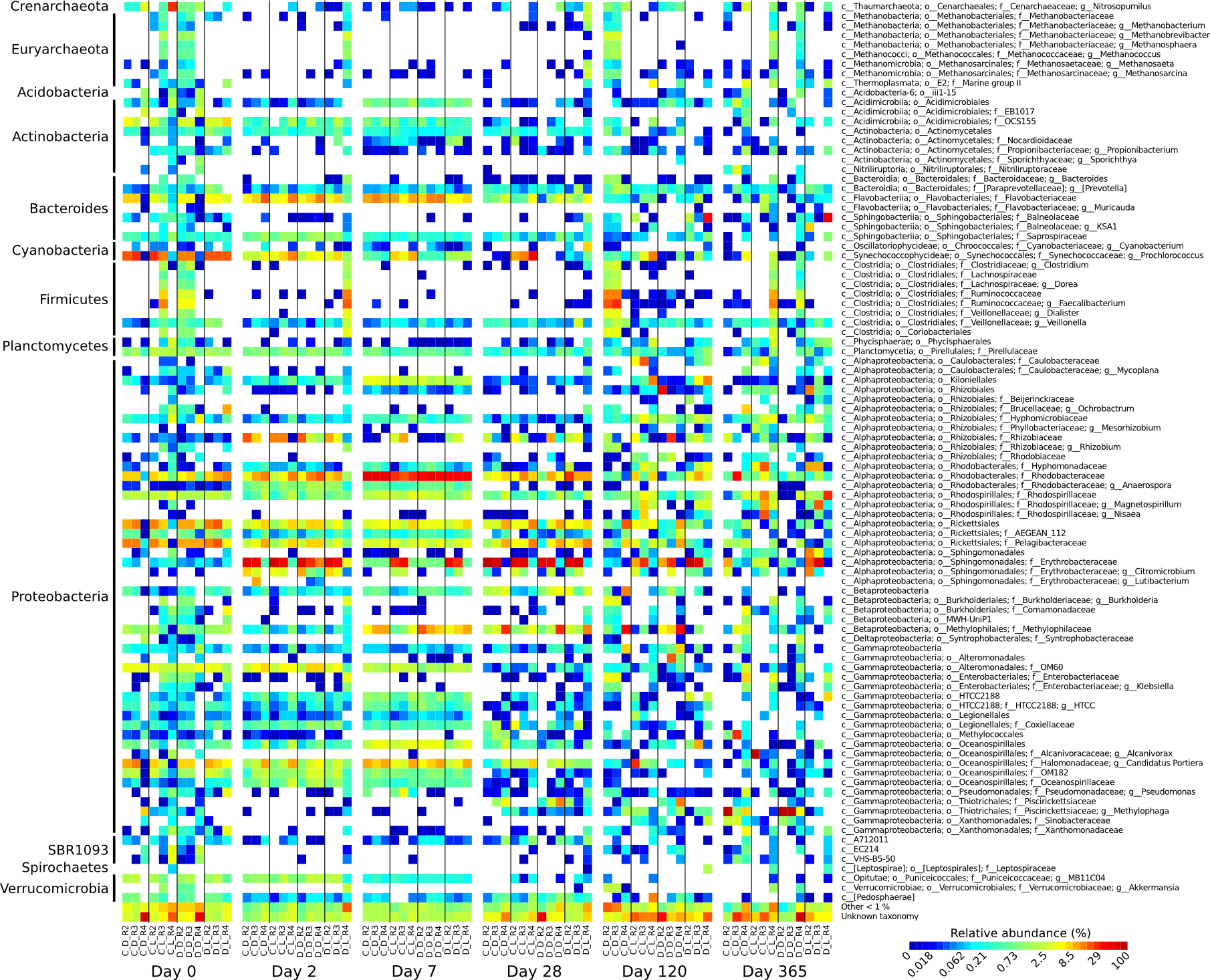

Supplement: Figure S4 — The four incubation conditions are control + dark (C_D), control + light (C_L), diuron + dark (D_D) and diuron + light (D_L), with each replicate shown (R2, R3 and R3). Only microbial genera reaching 1% are indicated. [file peerj-04-1758-s004.png]

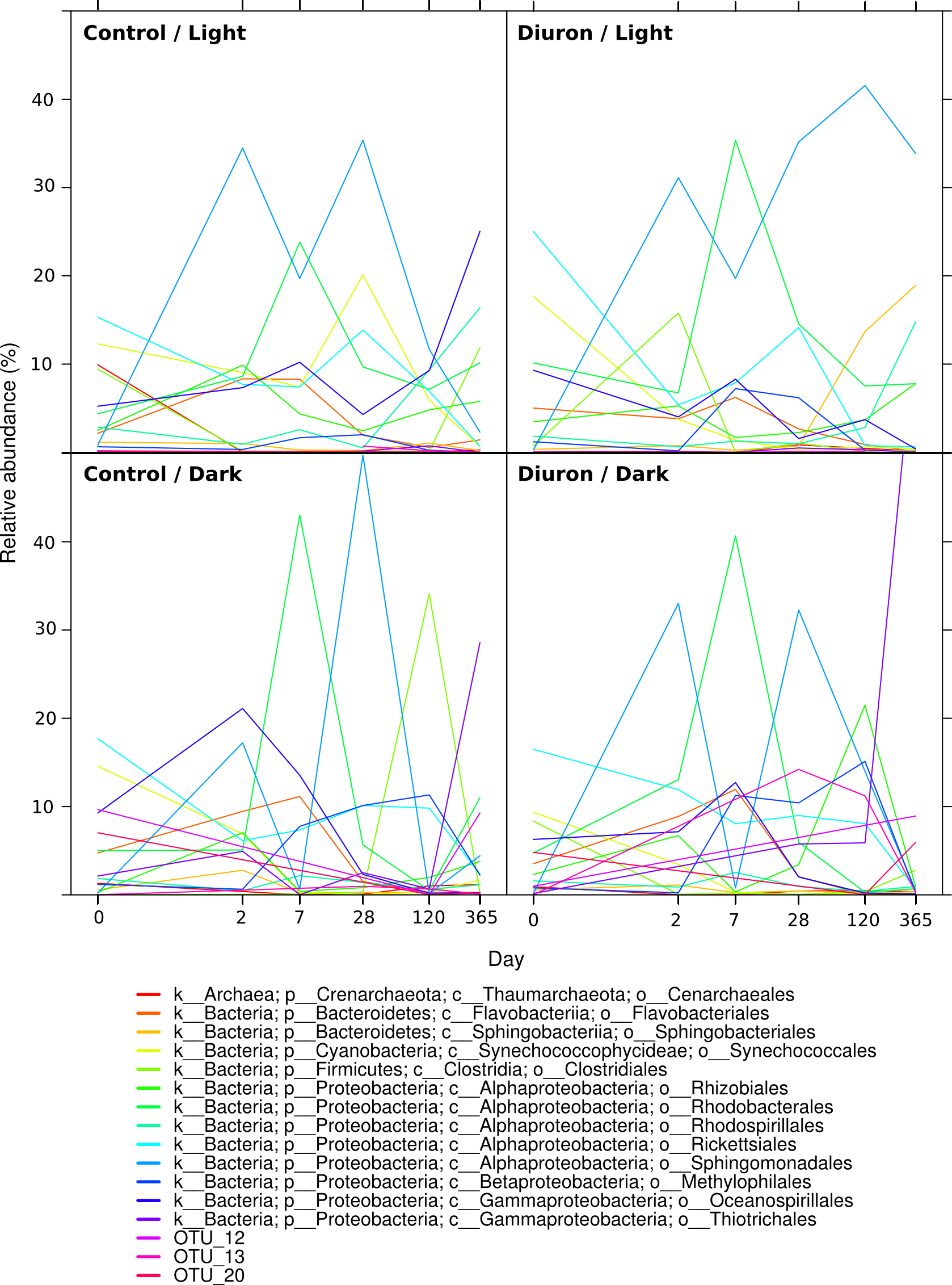

Supplement: Figure S5 — The three replicates in each of the four incubation conditions were averaged and only microbial orders reaching 10% are indicated. [file peerj-04-1758-s005.png]

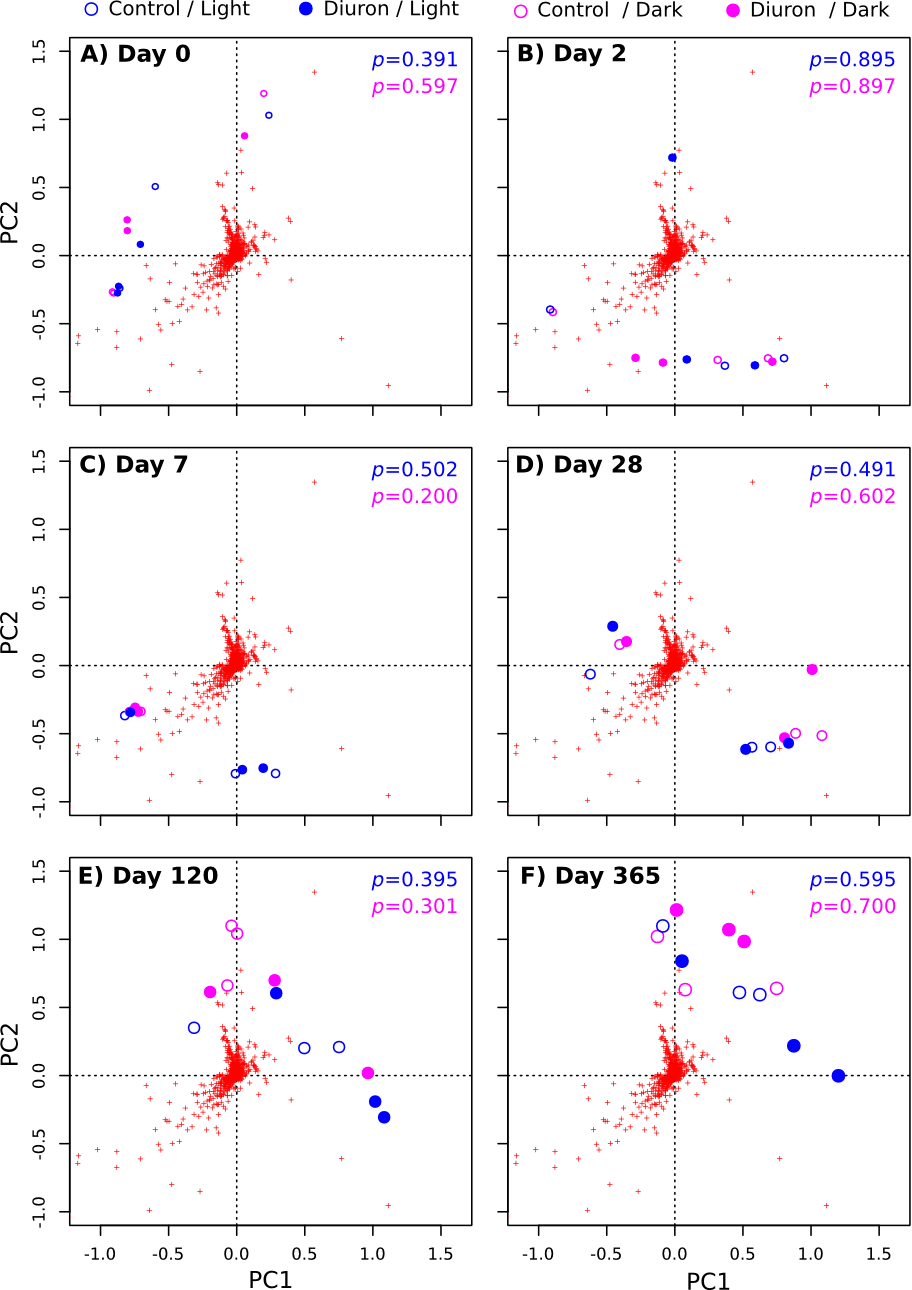

Supplement: Figure S6 — Circle size is proportional to sampling day. The p-values from PERMANOVA tests of the differences between diuron-treated and control incubations are shown. Red crosses indicate OTUs that drive sample differences. [file peerj-04-1758-s006.png]

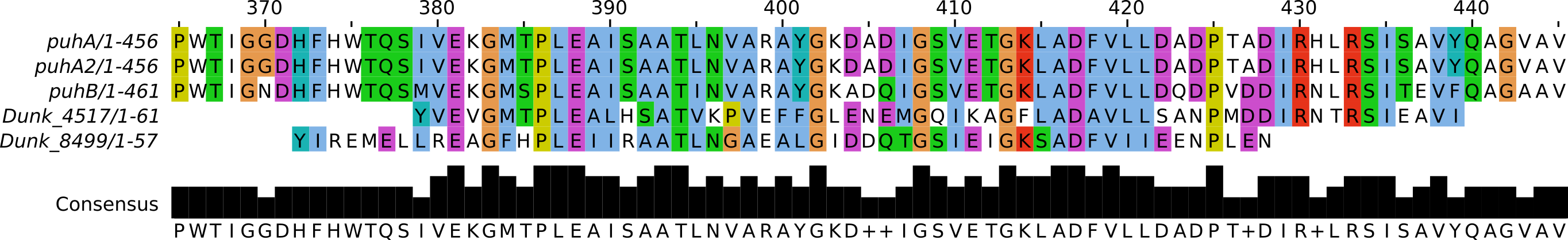

Supplement: Figure S7 — Residues are colored based on the Clustal X scheme. The bottom panel represents the number of conserved amino acids at each position and their consensus. [file peerj-04-1758-s007.png]
