# Supplementary material for: Diuron tolerance and potential degradation by pelagic microbiomes in the Great Barrier Reef lagoon
Source: PeerJ. 2016 Mar 8;4:e1758. doi: 10.7717/peerj.1758 (PMC4793316; doi:10.7717/peerj.1758)
Supplement: Supplementary file 2 [file peerj-04-1758-s008.odt]

| Treatment | Incubation length (days) | Replicate | # OTUS | Species richness (Chao1) | Species diversity (Shannon-Wiener) |
| --- | --- | --- | --- | --- | --- |
| Control / Light | 0 | R2 | 271 | 48.0 | 2.58 |
| R3 | 789 | 86.3 | 3.08 |
| R4 | 646 | 71.2 | 2.59 |
| 2 | R2 | 246 | 48.1 | 1.61 |
| R3 | 307 | 43.3 | 2.36 |
| R4 | 520 | 54.5 | 2.73 |
| 7 | R2 | 307 | 49.5 | 2.53 |
| R3 | 291 | 48.0 | 2.40 |
| R4 | 469 | 54.0 | 2.58 |
| 28 | R2 | 185 | 42.6 | 1.94 |
| R3 | 228 | 66.5 | 1.68 |
| R4 | 298 | 55.0 | 2.16 |
| 120 | R2 | 193 | 57.5 | 2.42 |
| R3 | 331 | 75.0 | 2.46 |
| R4 | 383 | 66.0 | 2.65 |
| 365 | R2 | 108 | 28.0 | 1.13 |
| R3 | 171 | 39.5 | 2.30 |
| R4 | 671 | 77.0 | 3.19 |
| Diuron / Light | 0 | R2 | 186 | 48.0 | 2.63 |
| R3 | 269 | 44.2 | 2.55 |
| R4 | 492 | 56.0 | 2.99 |
| 2 | R2 | 319 | 48.5 | 2.11 |
| R3 | 372 | 57.5 | 2.68 |
| R4 | 619 | 76.0 | 2.99 |
| 7 | R2 | 334 | 46.0 | 2.00 |
| R3 | 319 | 49.0 | 2.39 |
| R4 | 349 | 46.5 | 2.36 |
| 28 | R2 | 293 | 57.5 | 2.03 |
| R3 | 196 | 46.5 | 1.70 |
| R4 | 1061 | 100.5 | 2.92 |
| 120 | R2 | 165 | 44.4 | 1.19 |
| R3 | 286 | 49.0 | 2.08 |
| R4 | 257 | 55.4 | 2.19 |
| 365 | R2 | 218 | 53.8 | 2.36 |
| R3 | 167 | 33.5 | 1.73 |
| R4 | 259 | 56.4 | 1.86 |
| Control / Dark | 0 | R2 | 531 | 47.5 | 2.62 |
| R3 | 498 | 63.0 | 2.50 |
| R4 | 560 | 57.3 | 1.36 |
| 2 | R2 | 277 | 42.0 | 1.89 |
| R3 | 302 | 44.3 | 2.46 |
| R4 | 457 | 44.5 | 2.71 |
| 7 | R2 | 386 | 48.6 | 2.22 |
| R3 | 347 | 46.5 | 2.27 |
| R4 | 359 | 50.3 | 2.29 |
| 28 | R2 | 199 | 46.2 | 1.06 |
| R3 | 189 | 42.8 | 1.39 |
| R4 | 282 | 57.3 | 2.49 |
| 120 | R2 | 578 | 75.5 | 2.89 |
| R3 | 574 | 75.5 | 2.81 |
| R4 | 206 | 52.0 | 2.10 |
| 365 | R2 | 121 | 51.2 | 0.99 |
| R3 | 119 | 41.0 | 1.90 |
| R4 | 628 | 69.1 | 3.12 |
| Diuron / Dark | 0 | R2 | 964 | 85.3 | 3.22 |
| R3 | 936 | 81.2 | 3.14 |
| R4 | 634 | 57.5 | 1.99 |
| 2 | R2 | 256 | 47.8 | 1.86 |
| R3 | 375 | 58.5 | 2.66 |
| R4 | 360 | 47.0 | 2.73 |
| 7 | R2 | 300 | 58.0 | 2.36 |
| R3 | 337 | 53.2 | 2.21 |
| R4 | 350 | 50.0 | 2.26 |
| 28 | R2 | 73 | 31.0 | 0.94 |
| R3 | 191 | 49.5 | 1.98 |
| R4 | 428 | 57.0 | 2.46 |
| 120 | R2 | 114 | 47.0 | 1.58 |
| R3 | 168 | 45.0 | 2.08 |
| R4 | 305 | 65.0 | 2.28 |
| 365 | R2 | 63 | 33.3 | 0.28 |
| R3 | 111 | 60.0 | 0.38 |
| R4 | 918 | 82.0 | 1.91 |
