# Supplementary material for: Diuron tolerance and potential degradation by pelagic microbiomes in the Great Barrier Reef lagoon
Source: PeerJ. 2016 Mar 8;4:e1758. doi: 10.7717/peerj.1758 (PMC4793316; doi:10.7717/peerj.1758)
Supplement: Supplementary file 3 [file peerj-04-1758-s009.odt]

|  | RDP Classifier | Silva's SINA |
| --- | --- | --- |
| OTU_12 | Bacteria; Proteobacteria | Bacteria; Proteobacteria; Gammaproteobacteria; Salinisphaerales; Salinisphaeraceae; Salinisphaera |
| OTU_13 | Bacteria; Proteobacteria; Gammaproteobacteria | Bacteria; Proteobacteria; Gammaproteobacteria; Legionellales; Coxiellaceae; Coxiella |
| OTU_20 | Bacteria | Bacteria; Proteobacteria; Deltaproteobacteria; GR-WP33-30 |
