# Supplementary material for: Diuron tolerance and potential degradation by pelagic microbiomes in the Great Barrier Reef lagoon
Source: PeerJ. 2016 Mar 8;4:e1758. doi: 10.7717/peerj.1758 (PMC4793316; doi:10.7717/peerj.1758)
Supplement: Supplementary file 4 [file peerj-04-1758-s010.odt]

| Site | Upstream rivers | Distance to river mouth (km) | River discharge (ML/d) |
| --- | --- | --- | --- |
| Sampling week* | Dry season** | Wet season*** |
| Dunk Island | Tully | 14.8 | 1,050 | 7,600 | 16,000 |
| Herbert | 68.5 | 330 | 5,060 | 33,191 |
| Cape Ferguson | Haughton | 16.6 | 393 | 414 | 4,420 |
| Burdekin | 83.6 | 6,260 | 9,311 | 113,000 |
