# Supplementary material for: Diuron tolerance and potential degradation by pelagic microbiomes in the Great Barrier Reef lagoon
Source: PeerJ. 2016 Mar 8;4:e1758. doi: 10.7717/peerj.1758 (PMC4793316; doi:10.7717/peerj.1758)
Supplement: Supplementary file 5 [file peerj-04-1758-s011.odt]

| Site | Sampling week* | Dry season** | Wet season*** |
| --- | --- | --- | --- |
| Dunk Island | 28.0 | 26.5 | 30.9 |
| Cape Ferguson | 28.7 | 27.6 | 30.8 |
