# Supplementary material for: Diuron tolerance and potential degradation by pelagic microbiomes in the Great Barrier Reef lagoon
Source: PeerJ. 2016 Mar 8;4:e1758. doi: 10.7717/peerj.1758 (PMC4793316; doi:10.7717/peerj.1758)
Supplement: Supplementary file 6 [file peerj-04-1758-s012.odt]

| Site | Diuron | PSII HEq |
| --- | --- | --- |
| Maximum concentration (μg/L) | Frequency of detection (%) | Average value | Dry season | Wet season |
| Dunk Island | 19 | 92 | 2.9–3.8 | 5 | 4 |
| Cape Ferguson (Cape Cleveland & Magnetic Island) | 3.8 | 86–88 | 2.3–6.9 | 5 | 4-5 |
