# Supplementary material for: Diuron tolerance and potential degradation by pelagic microbiomes in the Great Barrier Reef lagoon
Source: PeerJ. 2016 Mar 8;4:e1758. doi: 10.7717/peerj.1758 (PMC4793316; doi:10.7717/peerj.1758)
Supplement: Supplementary file 7 [file peerj-04-1758-s013.odt]

| Site | Index year range | PN (μg/L) | PP (μg/L) | Chl a (μg/L) | SS (mg/L) | Secchi (m) | Turbidity (NTU) | Water quality index |
| --- | --- | --- | --- | --- | --- | --- | --- | --- |
| Dunk Island | 2007–10 | 1.08 | 0.11 | 0.49 | 2.39 | 5.39 | 2.37 | -0.10 |
| 2008–11 | 1.07 | 0.11 | 0.56 | 2.87 | 5.00 | 2.48 | -0.21 |
| 2009–12 | 1.08 | 0.11 | 0.54 | 2.33 | 4.68 | 2.79 | -0.16 |
| 2010–13 | 1.08 | 0.10 | 0.54 | 2.16 | 4.99 | 2.86 | -0.14 |
| Average | 1.08 | 0.11 | 0.53 | 2.44 | 5.02 | 2.63 | -0.15 |
| Cape Ferguson (Magnetic Island) | 2009–12 | 1.11 | 0.11 | 0.53 | 1.84 | 4.86 | 2.29 | -0.08 |
| 2010–13 | 1.07 | 0.11 | 0.52 | 1.85 | 4.98 | 2.64 | -0.10 |
| 2011–14 | 1.12 | 0.12 | 0.57 | 1.91 | 4.34 | 2.87 | -0.21 |
| Average | 1.10 | 0.11 | 0.54 | 1.87 | 4.73 | 2.60 | -0.13 |
