# Supplementary material for: Diuron tolerance and potential degradation by pelagic microbiomes in the Great Barrier Reef lagoon
Source: PeerJ. 2016 Mar 8;4:e1758. doi: 10.7717/peerj.1758 (PMC4793316; doi:10.7717/peerj.1758)
Supplement: Supplementary file 8 [file peerj-04-1758-s014.odt]

Supplementary Text

Meteorological and hydrological comparison of Dunk Island and Cape Ferguson

The similarity of the Dunk Island and Cape Ferguson samples, collected in October 2009 and May 2012 respectively, could not be assessed directly from their microbial composition due to the significant methodological differences between metagenomic and amplicon sequencing and analysis. However, a 3-year study of the microbial communities in the Great Barrier Reef lagoon (Angly et al., 2015) has revealed that community composition in this coastal area is driven by a combination of factors including exposure to river water, temperature and water quality. Environmental parameters available for the Dunk Island and Cape Ferguson areas provides a basis for the comparison of these two samples.

## Riverine exposure

Sites in the Great Barrier lagoon are exposed to varying amounts of riverine water, based on their distance to the nearest influencing river mouth and the magnitude of its freshwater discharge. River discharge increases rapidly in response to rainfall, with the majority of the rain occurring in the wet summer season (November through March) in this region.

The Dunk Island site is located in the Wet Tropics catchment and influenced by the nearby Tully River (~15 km) which was delivering over 1,000 ML/d of freshwater in the lagoon when sampling occurred, a number which is in agreement with the low discharge of this river in the dry season (Table S3). Over 60 km upstream of the Dunk Island site, the impact of the 330 ML/d flow of the Herbert River during the sampling week was negligible compared to that of the Tully River (Table S3).

In the neighboring Burdekin catchment, the Cape Ferguson site can be exposed to the waters of the Haughton and Burdekin rivers. The Haughton river mouth is only ~15 km away from the Cape Ferguson sampling site and discharged about a third as much as the Tully River at sampling time (Table S3). The discharge of the Burdekin River was about six times that of the Tully River during the sampling week, in agreement with its dry season discharge (Table S3). However the Burdekin River mouth is about six times as far away from Cape Ferguson as the Tully River mouth from Dunk Island.

The river discharge values were consistent with the low river flow characteristic of the dry season. Factoring in both distance to river mouth and river discharge, sampling sites seemed exposed to a comparable degree of riverine water.

# Temperature

Air temperature in the wet season is markedly higher than in the dry season. Records from the Bureau of Meteorology show that air temperature for Dunk Island and Cape Ferguson were close during the sampling week, at ~28-29 °C, consistent with seasonal averages (Table S4).

# Water quality

Water quality in the Great Barrier Reef lagoon is compromised by a combination of nutrients, sediments and herbicides brought by riverine floodwaters.

## Herbicide concentrations

Diuron is the main contributor (89%) to the photosystem II (PSII) herbicide-equivalent (HEq) index (Kennedy et al., 2012b), which combines the measured concentrations of several PSII-blocking herbicides. This index is classified into five categories based on the severity of its effects on marine organisms, from 1 for effects on the growth and death of aquatic plants and animals to 5 when no effects are demonstrated. Cape Ferguson is located between two sites (Cape Cleveland, 14.4 km upstream, and Magnetic Island, 26.2 km downstream) that were targeted along with Dunk Island for diuron and PSII HEq measurements between 2005 and 2010 (Lewis et al., 2009; Kennedy et al., 2012a).

Near Cape Ferguson, diuron was detectable in 87% of the samples and reached a maximum of 3.8 μg/L (Table S5). In comparison, diuron was detected 92% of the time at Dunk Island and peaked at 19 μg/L (Table S5). These values correspond to a classification in PSII HEq category 4 (reduced photosynthesis for two diatoms) in the wet season (yearly for Dunk Island, some years only for Cape Ferguson), and category 5 (no known effects) in the dry season (Table S5).

## Water quality index

An index of water quality for the GBR combining several individual water parameters (particulate organic nitrogen, particulate phosphorus, chlorophyll a suspended solids, Secchi depth and turbidity) was previously introduced (Thompson et al., 2013). This index ranges from -1 for very poor to +1 for very good and was calculated over for consecutive wet and dry seasons in the context of a long-term coral and water quality monitoring program (Thompson et al., 2014). The resulting values for the year 2009 at Dunk Island and 2012 at Cape Ferguson are reported in Table S6.

With the exception of suspended solids, which were more elevated by 30% at Dunk Island compared to near Cape Ferguson, all other reported water quality parameters were very close (Table S6). As a result, the water quality index for Dunk Island and Cape Ferguson was nearly identical at -0.14, corresponding to moderate water quality.

# Conclusions

Meteorological records confirmed that Dunk Island and Cape Fergus had low river discharge and air temperature at the time of sampling, as expected during dry summer. Long-term studies conducted around the sites demonstrated that their water quality and diuron exposure was similar (same water quality and PSII HEq classification). Microbial profiling in the GBR lagoon has shown that community composition does not show much spatial structuring in the dry season, when riverine input is low and water quality is high (relative to the wet season) (Angly et al., 2015). Given the data presented here, we therefore conclude that the Dunk Island and Cape Ferguson are comparable.

# Bibliography

Angly FE., Heath C., Morgan TC., Tonin H., Rich V., Schaffelke B., Bourne DG., Tyson GW. 2015. Marine microbial communities of the Great Barrier Reef lagoon are influenced by riverine floodwaters and seasonal weather events. PeerJ Accepted.

Kennedy K., Schroeder T., Shaw M., Haynes D., Lewis S., Bentley C., Paxman C., Carter S., Brando VE., Bartkow M., Hearn L., Mueller JF. 2012a. Long term monitoring of photosystem II herbicides – Correlation with remotely sensed freshwater extent to monitor changes in the quality of water entering the Great Barrier Reef, Australia. Marine Pollution Bulletin 65:292–305.

Kennedy K., Devlin M., Bentley C., Lee-Chue K., Paxman C., Carter S., Lewis SE., Brodie J., Guy E., Vardy S., Martin KC., Jones A., Packett R., Mueller JF. 2012b. The influence of a season of extreme wet weather events on exposure of the World Heritage Area Great Barrier Reef to pesticides. Marine Pollution Bulletin 64:1495–1507.

Lewis SE., Brodie JE., Bainbridge ZT., Rohde KW., Davis AM., Masters BL., Maughan M., Devlin MJ., Mueller JF., Schaffelke B. 2009. Herbicides: A new threat to the Great Barrier Reef. Environmental Pollution 157:2470–2484.

Thompson A., Schaffelke B., Logan M., Costello P., Davidson J., Doyle J., Furnas M., Gunn K., Liddy M., Skuza M., Uthicke S., Wright M., Zagorskis I. 2013. Reef Rescue Marine Monitoring Program. Annual report of AIMS activities 2012 to 2013 - Inshore water quality and coral reef monitoring. Townsville: Australian Institute of Marine Science.

Thompson A., Lønborg C., Costello P., Davidson J., Logan M., Furnas M., Gunn K., Liddy M., Skuza M., Uthicke S., Wright M., Zagorskis I. 2014. Marine Monitoring Program. Annual Report of AIMS Activities 2013 to 2014– Inshore water quality and coral reef monitoring. Townsville: Australian Institute of Marine Science.
